# A stromal platform for robust expansion of functional IL-10–producing B cells for immune regulation

**DOI:** 10.1172/jci.insight.197393

**Published:** 2026-04-22

**Authors:** Ryo Kawakami, Keisuke Imabayashi, Akemi Baba, Yuichi Saito, Kazuhiko Kawata, Yutaro Yada, Airi Shibata, Rinka Ito, Ryo Kurasawa, Ryota Higuchi, Sungyeon Park, Hiroaki Niiro, Shinya Tanaka, Yoshihiro Baba

**Affiliations:** 1 Division of Immunology and Genome Biology, Medical Institute of Bioregulation, Kyushu University, Fukuoka, Japan.; 2 Department of Otorhinolaryngology, Faculty of Medical Sciences,; 3 Department of Pediatrics, Faculty of Medical Sciences, and; 4 Department of Medical Education, Faculty of Medical Sciences, Kyushu University Graduate School of Medical Sciences, Fukuoka, Japan.

**Keywords:** Autoimmunity, Immunology, B cells

## Abstract

IL-10–producing B cells exert immunosuppressive effects, yet their low abundance and poor in vitro viability have limited their therapeutic application. Here, we developed a stromal coculture system using MS5 cells engineered to express human CD40L, BAFF, and IFN-β1 (MS5-3F, for “3 factors”), which enables robust induction and greater than 1000-fold expansion of human IL-10–producing B cells. The expanded cells showed phenotypic and transcriptional profiles characteristic of unswitched (IgM^+^) plasmablasts and potently suppressed CD4^+^ T cell proliferation in an IL-10–dependent manner. MS5-3F–expanded B cells also increased the frequency of regulatory T cells in vitro, an effect that was not abrogated by IL-10/IL-10R blockade, suggesting contributions from additional mechanisms. IL-10 production originated predominantly from naive B cells, rather than memory B cells. Furthermore, B cells from patients with systemic lupus erythematosus, despite impaired IL-10 production under conventional conditions, were efficiently differentiated into IL-10–producing B cells using this system. The expanded cells showed minimal IgG-secreting output. Our platform offers a scalable strategy for generating human regulatory B cells, laying the foundation for B cell–based immunotherapies.

## Abstract 2

A stromal co-culture platform enables robust expansion of functional IL-10–producing B cells from both healthy individuals and SLE patients, offering new avenues for B cell–based immunotherapies.

## Introduction

Autoimmune diseases, characterized by aberrant immune responses against self-antigens, are a diverse group of chronic conditions. Despite therapeutic advances, options remain limited due to an incomplete understanding of the immunological mechanisms driving disease progression (1). Among the various immune cells implicated in immunity, B cells contribute critically to autoimmune pathology via autoantibody production, antigen presentation, and cytokine secretion (2). While self-reactive memory B cells and plasma cells are widely recognized for their pathogenic roles, particular attention has recently focused on age-associated B cells (ABCs), a subset that accumulates with age and exacerbates autoimmune pathology (3, 4). In contrast, a different subset, termed regulatory B cells (Bregs), exerts immunosuppressive effects primarily through the production of interleukin-10 (IL-10), and serves as key modulator of inflammation and autoimmunity (5, 6). In murine models, several IL-10–producing B cell subsets have been described, including CD1d^hi^CD5^+^ B10 cells, T2-MZP transitional cells, and IL-10–producing plasmablasts or plasma cells (7–10). These subsets mitigate disease severity in models of lupus, collagen-induced arthritis (CIA), experimental autoimmune encephalomyelitis (EAE), and type 1 diabetes mellitus (T1DM), primarily via IL-10–dependent suppression of pathogenic immune responses (8, 9, 11–14). In humans, patients with autoimmune diseases such as systemic lupus erythematosus (SLE), systemic sclerosis, rheumatoid arthritis, multiple sclerosis, and T1DM have been shown to exhibit reduced frequencies and impaired function of IL-10–producing B cell populations (11, 15–19). This impairment correlates with disease activity, suggesting that enhancing IL-10–producing B cell function may have therapeutic potential (2, 20, 21).

Despite growing evidence of their importance, the molecular pathways governing IL-10–producing B cell differentiation remain poorly defined. While extrinsic stimuli, such as CpG-induced TLR9 activation, CD40L, IL-21, and type I IFNs, act synergistically to induce IL-10 production (9, 22–25), at the intracellular level, transcriptional regulators such as IRF4, NFAT, AhR, MAF, and STAT3 contribute to IL-10 transcriptional regulation (26–29). However, the integrated mechanisms by which these signals drive the differentiation and expansion of functional IL-10–producing B cells remain incompletely defined, particularly in the human setting. These limited mechanistic insights, combined with the inherently low frequency and poor in vitro viability of IL-10–producing B cells, present substantial challenges for their detailed study and therapeutic exploitation. Although several protocols, such as CpG/CD40L stimulation, metabolic modulation, and cytokine cocktails (30, 31), have been explored, few studies have successfully achieved the in vitro expansion and long-term maintenance of IL-10–producing B cells (32, 33). We previously demonstrated that type I IFN promotes the differentiation of IL-10–producing plasmablasts (9); however, they failed to expand under conventional liquid culture conditions. Thus, a platform that can support both robust induction and sustained expansion of IL-10–producing B cells remains an unmet need for therapeutic development.

In this study, we developed a stromal coculture system using MS5 cells engineered to express human CD40L, BAFF, and IFN-β1. This innovative culture platform robustly induces the expansion of human IL-10–producing B cells, while predominantly generating unswitched (IgM^+^) plasmablasts. Crucially, the expanded B cells exhibited potent immunosuppressive activity, effectively suppressing CD4^+^ T cell proliferation through IL-10–dependent mechanisms. Moreover, the system supported the differentiation of IL-10–producing B cells from SLE patient–derived cells, similar to healthy control donors (HCs). Thus, this platform enables the generation of functional IL-10–producing B cells and provides a foundation for B cell–based immunotherapies in autoimmune diseases.

## Results

### Efficient induction and expansion of human IL–10–producing B cells using engineered stromal cells.

**Figure 1:**
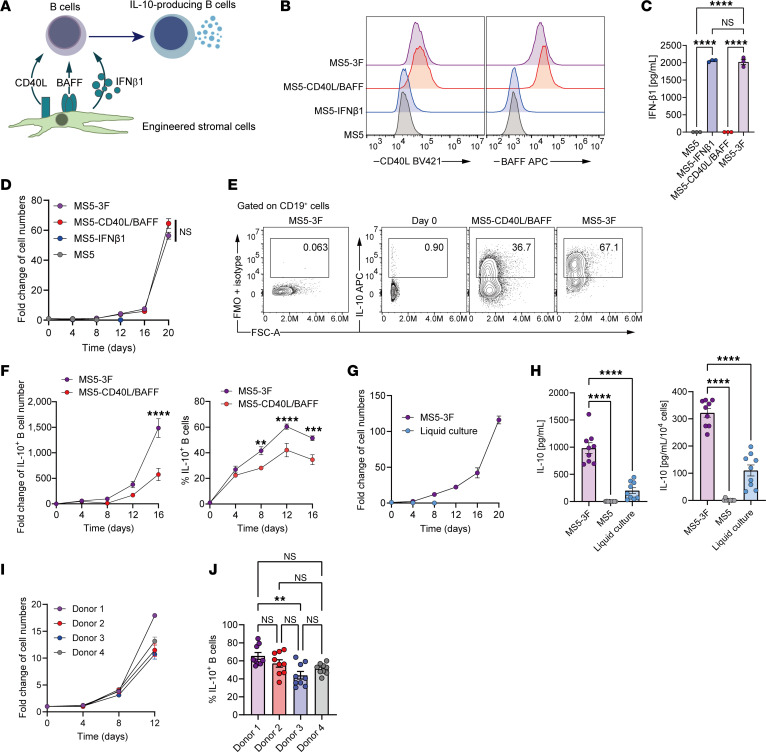
Establishment of a stromal coculture system for human IL-10–producing B cell induction and expansion. (**A**) Schematic overview of the coculture system. MS5 stromal cells were genetically engineered to express human IFN-β1 (MS5-IFNβ1), CD40L and BAFF (MS5-CD40L/BAFF), or all 3 factors (MS5-3F), to support the differentiation of peripheral blood B cells into IL-10–producing B cells. (**B**) Representative flow cytometry histograms of human CD40L and BAFF expressed on MS5 lines. See also Supplemental Figure 1. (**C**) Quantification of IFN-β1 production by MS5 lines. (**D**) Fold expansion of total B cells cocultured with different MS5 lines in the presence of CpG ODN. Cell numbers were assessed every 4 days and normalized to the initial input to calculate fold change. See also Supplemental Figure 2A. (**E** and **F**) Induction and expansion of IL-10–producing B cells cocultured with MS5-CD40L or MS5-3F. (**E**) Representative flow cytometry plots of IL-10–producing B cells on days 0 and 12 of coculture, assessed using IL-10 secretion assay. (**F**) Fold expansion and temporal changes in frequencies of IL-10^+^ cells. (**G** and **H**) Comparison of B cell expansion and IL-10 production between MS5-3F coculture and conventional liquid culture conditions. (**G**) Fold expansion of total B cells. (**H**) IL-10 secretion in culture supernatants on day 4 assessed by ELISA. (**I**) Expansion of B cells from multiple healthy donors in MS5-3F coculture. (**J**) Summarized graph of percentage IL-10 in B cells from different donors on day 12 of MS5-3F coculture. See also Supplemental Figure 2E. Data from 3 independent experiments were combined (**C**, **D**, and **F**: *n* = 3; **G**–**J**: *n* = 9). Data are presented as mean ± SEM. *P* values are from 1-way ANOVA with Tukey’s post hoc test (**C**, **H**, and **J**) or 2-way (**D** and **F**) ANOVA with Bonferroni’s post hoc test. **P ≤ 0.01; ***P ≤ 0.001; ****P ≤ 0.0001. NS, not significant.

To establish a method for efficiently inducing and expanding human IL-10–producing B cells in vitro, we engineered stromal MS5 cells to express human IFN-β1 (MS5-IFNβ1), CD40L and BAFF (MS5-CD40L/BAFF), or all 3 factors simultaneously (MS5-3F) (Figure 1A). The expression of each factor was confirmed by flow cytometry (CD40L, BAFF) and ELISA of culture supernatants (IFN-β1) (Figure 1, B and C, and Supplemental Figure 1; supplemental material available online with this article; https://doi.org/10.1172/jci.insight.197393DS1). Peripheral blood B cells were cocultured on each stromal line (Supplemental Figure 2A). Total B cell numbers increased robustly only in MS5-CD40L/BAFF and MS5-3F cocultures (Figure 1D). We therefore compared their capacity to secrete IL-10 under these 2 conditions. Using a single-cell IL-10 secretion assay, we found that MS5-3F coculture yielded significantly higher frequencies of IL-10–producing B cells, suggesting a synergistic effect of IFN-β with CD40L and BAFF (Figure 1, E and F). IL-10^+^ B cells peaked around day 12 and maintained stable frequencies thereafter. Notably, the system achieved a 1200-fold expansion of IL-10–producing B cells by day 16 (Figure 1F). To optimize culture conditions, we assessed the requirement for CpG, IL-2, and IL-6. CpG was essential for robust proliferation (Supplemental Figure 2B), while IL-2 and IL-6 had no additive effect (Supplemental Figure 2, C and D). In contrast to liquid culture with CpG, IL-2, IL-6, and IFN-α, which led to rapid cell loss, the MS5-3F coculture supported sustained viability and approximately 100-fold expansion (Figure 1G). ELISA further confirmed enhanced IL-10 secretion in the MS5-3F system (Figure 1H). Finally, consistent results were observed across multiple donors, demonstrating the platform’s robustness and broad applicability (Figure 1, I and J, and Supplemental Figure 2E).

### Phenotypic characterization of human B cells expanded in stromal coculture.

**Figure 2:**
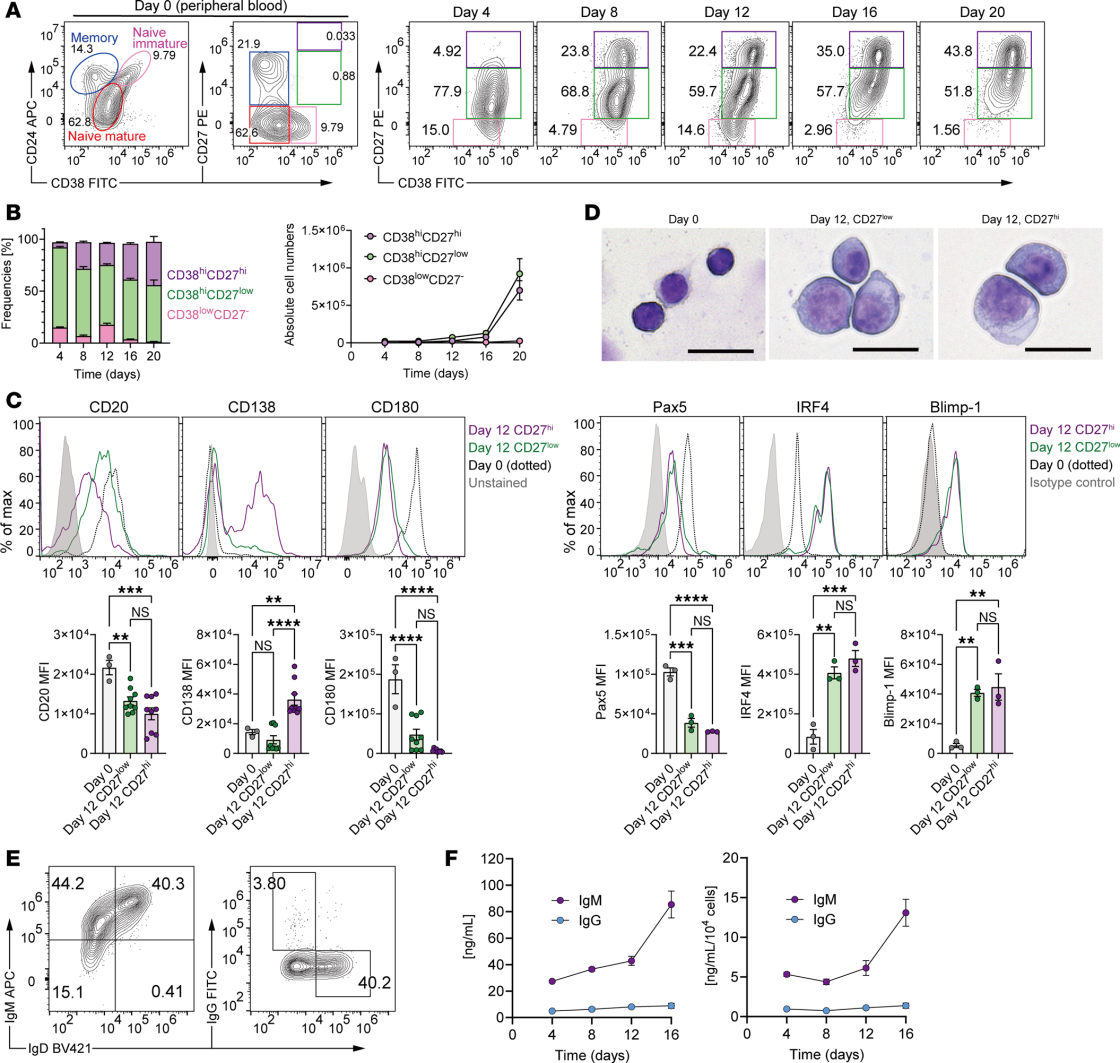
MS5-3F coculture induces IgM^+^ plasmablast differentiation from human B cells. (**A** and **B**) Temporal changes in activation markers of B cells cocultured with MS5-3F. (**A**) Representative flow cytometry plots. (**B**) Frequencies and absolute cell numbers of indicated subsets among CD19^+^ cells. (**C**) Representative histograms and summarized MFI of surface markers and transcriptional factors expressed on CD27^hi^ and CD27^lo^ B cells on days 0 and 12 of MS5-3F coculture. See also Supplemental Figure 3. (**D**) Morphological analysis of primary B cells (day 0) and MS5-3F–induced B cell subsets, stained with May-Grünwald-Giemsa. CD27^hi^ and CD27^lo^ B cells were sorted on day 12 of coculture. Original magnification, ×400. Scale bars: 20 μm. (**E**) Representative flow cytometry plots of IgM, IgD, and IgG on B cells on day 12 of MS5-3F coculture. (**F**) Quantification of IgM and IgG antibody production by cocultured B cells. Culture supernatants were harvested every 4 days and analyzed by ELISA. Data from 3 independent experiments were combined (**B** and **C**: *n* = 3 or 9; **F**: *n* = 9). Data are presented as mean ± SEM. *P* values are from 1-way ANOVA with Tukey’s post hoc test (**C**). **P ≤ 0.01; ***P ≤ 0.001; ****P ≤ 0.0001. NS, not significant.

To characterize the nature of B cells induced in MS5-3F coculture, we assessed surface markers associated with B cell differentiation. Flow cytometry identified major subsets based on CD24, CD38, and CD27: naive mature B cells (CD24^+^CD38^–^CD27^–^), naive immature B cells (CD24^hi^CD38^lo^CD27^–^), memory B cells (CD24^hi^CD38^–^CD27^+^), and plasmablasts/plasma cells (CD38^hi^CD27^lo^ or CD38^hi^CD27^hi^) (Figure 2, A and B). Prior to coculture (day 0), the population consisted mainly of naive mature, naive immature, and memory B cells. By day 4, most cells adopted a CD38^+^ phenotype. By day 12, 60%–80% of cells acquired a CD38^hi^CD27^lo^ phenotype, previously associated with IL-10–producing plasmablasts (9). Subsequently, these cells transitioned to a CD38^hi^CD27^hi^ phenotype. Although the frequency of CD38^hi^CD27^hi^ cells seemed to increase over time, absolute count analyses showed simultaneous increases in both CD38^hi^CD27^lo^ and CD38^hi^CD27^hi^ cells (through day 20), indicating ongoing accumulation and maturation rather than just loss of CD38^hi^CD27^lo^ cells. Further analysis revealed that both subsets downregulated CD180 and Pax5 and upregulated IRF4 and Blimp-1 (Figure 2C). Rapid and pronounced changes in these transcription factors were evident as early as day 4 (Supplemental Figure 3). CD138 expression was detected in CD38^hi^CD27^hi^ cells but absent in CD38^hi^CD27^lo^ cells, confirming that the former represented mature plasma cells, whereas the latter corresponded to plasmablasts. Morphologically, these cells exhibited features characteristic of antibody-secreting cells, including larger size, abundant cytoplasm, and eccentric nuclei (Figure 2D). These observations support the notion that CD38^hi^CD27^lo^ and CD38^hi^CD27^hi^ subsets represent sequential stages along the plasmablast-to–plasma cell differentiation axis.

IL-10 has been reported to act in an autocrine manner on human B cells to promote plasmablast differentiation (34). To address this, we assessed B cell differentiation on MS5-3F in the presence or absence of IL-10– and IL-10R–blocking antibodies. IL-10 pathway blockade significantly reduced the overall number of B cells, whereas the frequencies of CD38^hi^CD27^lo^ and CD38^hi^CD27^hi^ populations were unchanged under these conditions (Supplemental Figure 4). Thus, autocrine IL-10 is functionally active and primarily supports the expansion and/or survival of plasmablast/plasma cell populations, rather than altering their relative distribution.

Next, we examined the immunoglobulin isotype profile of the expanded cells. The majority of cells expressed IgM, while IgG^+^ cells were rare, despite some loss of IgD in a portion of the population (Figure 2E). ELISA confirmed increasing IgM secretion over time, with minimal IgG detected (Figure 2F). Thus, the MS5-3F coculture system supports the expansion of unswitched (IgM^+^) plasmablasts, with minimal IgG^+^ cells.

### Phenotypic characterization of human IL-10–producing B cells in stromal coculture.

**Figure 3:**
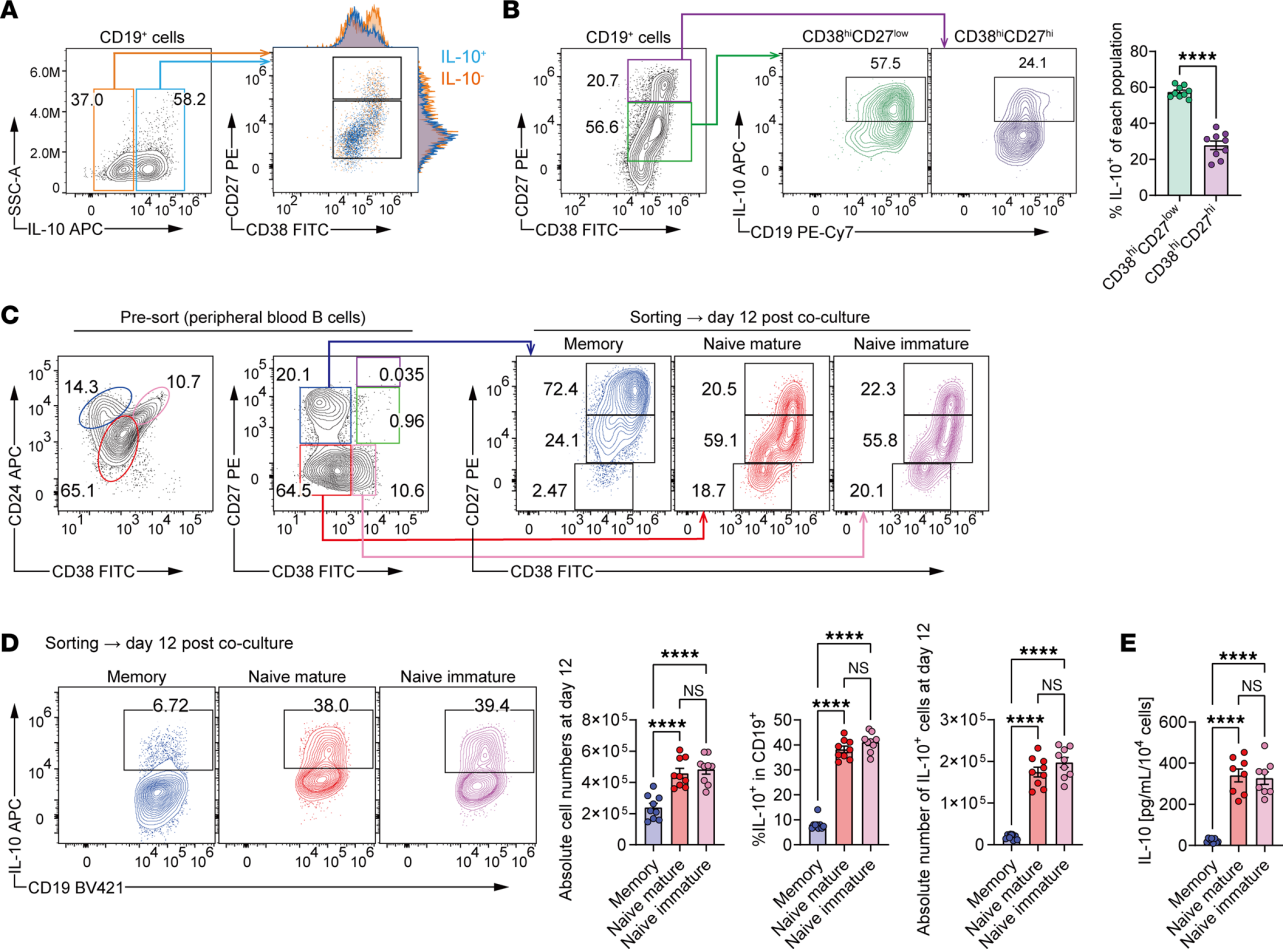
Phenotype of MS5-3F–induced IL-10–producing B cells. (**A** and **B**) Phenotypic comparison of IL-10^+^ and IL-10^–^ B cells on day 12 of MS5-3F coculture. (**A**) Comparison of CD27 and CD38 expression between IL-10^+^ and IL-10^–^ fractions. (**B**) Frequencies of IL-10^+^ cells within CD27^hi^ and CD27^lo^ subsets. See also Supplemental Figure 6. (**C**) Representative flow cytometry plots of activation markers expressed on peripheral blood B cells, and sorted memory, naive mature, and naive immature subsets on day 12 of MS5-3F coculture. Sorting strategy is shown in Supplemental Figure 6. (**D**) IL-10 production by each sorted B cell population on day 12 of MS5-3F coculture. (**E**) ELISA of IL-10 secreted by each sorted B cell population on day 12 of MS5-3F coculture. Data from 3 independent experiments were combined (**B** and **D**: *n* = 9; **E**: *n* = 8). Data are presented as mean ± SEM. *P* values are from 2-tailed, unpaired Student’s *t* test (**B**) and 1-way ANOVA with Tukey’s post hoc test (**D** and **E**). ****P ≤ 0.0001. NS, not significant.

To clarify which subset of differentiated B cells mainly produces IL-10, we performed IL-10 secretion assays in conjunction with phenotypic gating. At both day 12 and day 20, IL-10^+^ cells were distributed across both CD38^hi^CD27^lo^ and CD38^hi^CD27^hi^ subsets, with a higher frequency observed in the former (Figure 3, A and B, and Supplemental Figure 5). To trace their origin, B cells were sorted into naive immature, naive mature, and memory subsets before MS5-3F coculture (Supplemental Figure 6). Immature and mature B cells efficiently differentiated into CD38^hi^CD27^lo^ plasmablasts, while memory B cells showed limited differentiation, predominantly giving rise instead to CD38^hi^CD27^hi^ cells (Figure 3C). Consistently, flow cytometric assays and ELISAs showed that naive-derived plasmablasts produced higher levels of IL-10 than memory-derived counterparts (Figure 3, D and E). These findings indicate that IL-10–producing B cells in this system are primarily plasmablasts, which arise predominantly from naive B cells.

To characterize the functional polarization of MS5-3F–expanded IL-10^+^ B cells, we next assessed whether they coproduce pro- or antiinflammatory cytokines. Intracellular cytokine staining showed that IFN-γ and IL-12 were undetectable in IL-10^+^ cells, whereas a fraction of IL-10^+^ B cells coproduced IL-6 (~40%) or TNF-α (~3%). The coexistence of a subset of IL-10^+^ B cells with proinflammatory cytokines is consistent with the reported heterogeneity of human IL-10–producing B cells (21, 35) (Supplemental Figure 7). We also examined additional antiinflammatory mediators and found that TGF-β was detectable in approximately 10% of IL-10^+^ cells, while granzyme B was undetectable by flow cytometry; IL-35 was not detectable in IL-10^+^ cells by ELISA under our culture conditions. Overall, cytokine profiling suggested an IL-10–skewed phenotype, with only a limited fraction of IL-10^+^ cells coproducing proinflammatory cytokines.

To further assess the migratory potential of MS5-3F–expanded IL-10^+^ B cells, we examined the expression of representative chemokine receptors and adhesion molecules by flow cytometry (Supplemental Figure 8). IL-10^+^ cells showed detectable expression of CXCR3, CXCR4, and CXCR5, whereas CCR2, CCR5, and CCR7 were undetectable or minimal under our conditions. In parallel, IL-10^+^ cells broadly expressed adhesion molecules implicated in tissue entry and retention, including VLA-4 (integrin α4/CD49d and β1/CD29), ICAM-1, CD44, and CD62L (L-selectin). Collectively, this profile is compatible with trafficking to inflamed peripheral tissues (CXCR3 together with VLA-4/ICAM-1) while retaining potential access to secondary lymphoid environments (CXCR5/CXCR4 and CD62L). These data provide a phenotypic framework for considering where MS5-3F–expanded IL-10^+^ B cells may localize and regulate T cells in vivo.

To assess the robustness of IL-10 expression under inflammatory cues, MS5-3F–induced IL-10^+^ B cells were harvested and recultured for 4 days in the presence of a proinflammatory cytokine cocktail under a high-dose condition and at serial dilutions (36–38). Even at the highest dose, viability and the frequency of IL-10^+^ cells among viable B cells showed only a slight reduction, whereas lower-dose conditions had minimal impact on viability and IL-10 expression (Supplemental Figure 9, A and B). Overall, IL-10 expression was largely preserved across conditions, supporting the robustness of the MS5-3F–induced IL-10 program under inflammatory cytokine exposure.

### MS5-3F–induced human IL-10–producing B cells suppress T cell activation.

**Figure 4:**
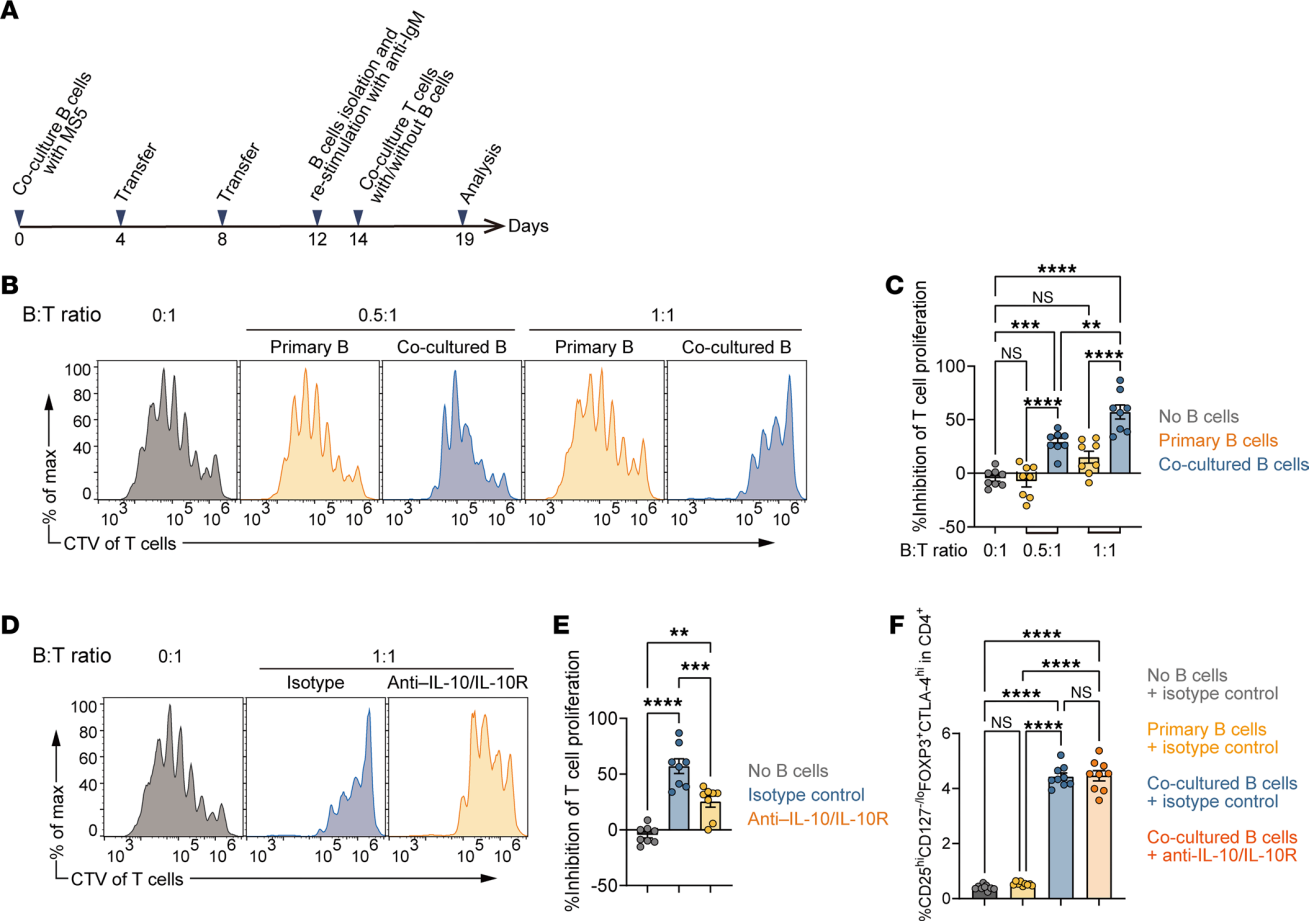
Suppressive function of MS5-3F–induced IL-10–producing B cells. (**A**) Schematic of the T cell suppression assay. (**B** and **C**) Suppression of T cell proliferation by cocultured B cells. (**B**) Representative CTV dilution histograms of CD4^+^ T cells cocultured with either primary B cells or MS5-3F–induced B cells at various B/T ratios. (**C**) Summarized percentage inhibition of T cell proliferation. (**D** and **E**) IL-10–dependent suppression of T cell proliferation. (**D**) Representative CTV dilution histograms of CD4^+^ T cells cocultured with MS5-3F–induced B cells in the presence of anti–IL-10/anti–IL-10R neutralizing antibodies or isotype controls. (**E**) Summarized percentage inhibition. (**F**) Summarized graphs showing Treg induction by primary B cells and MS5-3F–induced cocultured B cells. Peripheral blood–derived CD4^+^CD25^–^ non-Tregs were cocultured with primary B cells or MS5-3F–induced B cells (cocultured with MS5-3F for 12 days and restimulated for 2 days) on plate-bound anti-CD3 antibody for 3 days. To evaluate the role of IL-10 in Treg induction, anti–IL-10/anti–IL-10R monoclonal antibodies or isotype control were added during the culture. Tregs were defined as CD4^+^CD25^hi^CD127^–/lo^CTLA4^hi^FOXP3^+^ cells. See also Supplemental Figure 10C for representative flow cytometry plots. Data from 3 independent experiments were combined (**C** and **E**: *n* = 8, **F**: *n* = 9). Data are presented as mean ± SEM. *P* values are from 1-way ANOVA with Tukey’s post hoc test (**C**, **E**, and **F**). **P ≤ 0.01; ***P ≤ 0.001; ****P ≤ 0.0001. NS, not significant.

To evaluate the immunosuppressive function of IL-10–producing B cells expanded in MS5-3F coculture, we examined their ability to suppress CD4^+^ T cell proliferation in vitro (Figure 4A). B cells were cultured with MS5-3F until day 12 and then restimulated with anti-IgM antibodies to enhance IL-10 production. Restimulated B cells were then cocultured with autologous naive CD4^+^CD25^–^ T cells activated by anti-CD3/anti-CD28 antibodies, and T cell proliferation was assessed by CellTrace Violet (CTV) dilution. Freshly isolated B cells had no effect, whereas MS5-3F–induced B cells potently inhibited T cell proliferation in a dose-dependent manner (Figure 4, B and C). In parallel, we compared the suppressive effectiveness of IL-10^+^ B cells induced by MS5-3F with autologous CD4^+^CD25^+^ regulatory T cells (Tregs) using the same CTV assay. Under our conditions, the percentage inhibition achieved by these IL-10^+^ B cells was comparable to that of Tregs (Supplemental Figure 10, A and B). Blockade of IL-10 with neutralizing antibodies against IL-10 and IL-10R substantially diminished the suppressive effect of the MS5-3F–induced B cells, indicating that inhibition of CD4^+^ T cell proliferation is largely dependent on IL-10 (Figure 4, D and E). Given prior reports that human Bregs can promote Treg induction/expansion (21, 39), we next assessed whether these cells also promote Treg expansion. MS5-3F–cocultured B cells significantly increased the frequency of CD4^+^CD25^hi^CD127^–/lo^Foxp3^+^CTLA4^hi^ Tregs compared with cultures containing freshly isolated B cells (Figure 4F and Supplemental Figure 10C). Notably, IL-10/IL-10R blockade did not abrogate this increase, suggesting that Treg expansion occurs largely independently of IL-10 signaling under these conditions.

### Induction of IL-10–producing B cells from patients with SLE in MS5-3F coculture.

**Figure 5:**
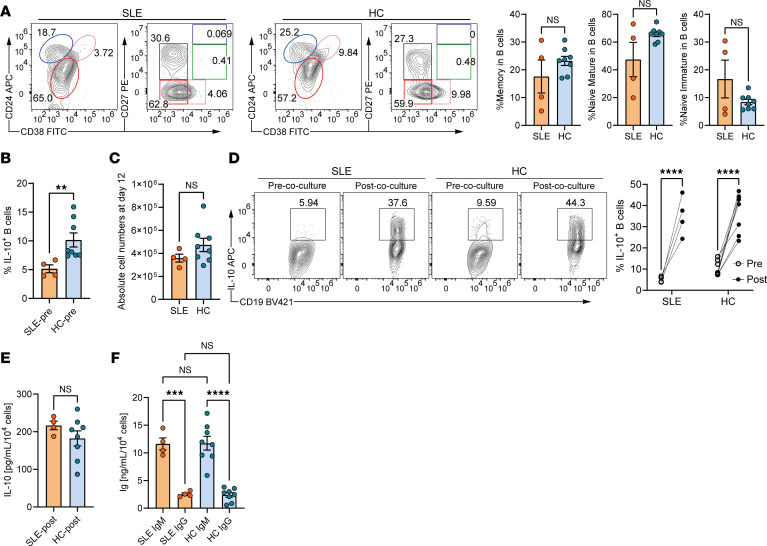
Induction of IL-10–producing B cells from patients with SLE and healthy donors in MS5-3F coculture. (**A**) Representative flow cytometry plots of surface markers expressed on B cells from patients with SLE and healthy controls (HCs), and summarized frequencies of memory (CD38^–^CD27^+^), naive mature (CD38^–^CD27^–^), and naive immature (CD38^lo^CD27^–^) B cells among CD19^+^ cells. See also Supplemental Table 1. (**B**) Summarized frequencies of IL-10^+^ B cells from patients with SLE and HCs stimulated with CD40L for 2 days (pre-co-culture). B cell stimulation was performed using CD40L-expressing MS5 cells. For the construction of the MS5 cells, see Supplemental Figure 11. (**C** and **D**) Induction and expansion of IL-10^+^ B cells from patients with SLE and HCs. (**C**) Fold expansion of total B cells from patients with SLE and HCs cocultured with MS5-3F for 12 days. (**D**) Representative flow cytometry plots and summarized frequencies of IL-10^+^ B cells from SLE and HC B cells, stimulated with CD40L for 2 days (pre-co-culture) or cocultured with MS5-3F for 12 days (post-co-culture). (**E**) ELISA of IL-10 secreted by SLE and HC B cells cocultured with MS5-3F for 12 days. (**F**) ELISA of IgM and IgG secreted by SLE and HC B cells cocultured with MS5-3F for 12 days. Data from 2 independent experiments were combined (SLE: *n* = 4; HC: *n* = 8). Data are presented as mean ± SEM. *P* values are from 2-tailed, unpaired Welch’s *t*-test (**A**–**E**) and 1-way ANOVA with Tukey’s post hoc test (**F**). **P ≤ 0.01; ***P ≤ 0.001; ****P ≤ 0.0001. NS, not significant.

Previous studies have reported that B cells from patients with SLE exhibit impaired IL-10 production in specific regulatory subsets, particularly CD24^hi^CD38^hi^ naive immature B cells, in response to CD40 stimulation (11). To determine whether our MS5-3F coculture system can induce IL-10–producing B cells from SLE patient–derived B cells, we performed parallel cultures with cells from SLE patients and HCs. Flow cytometric analysis of baseline B cell composition showed no significant differences in the proportions of naive and memory subsets between SLE and HC samples, although donor variability was observed, potentially reflecting differences in disease activity (Figure 5A and Supplemental Table 1). As expected, SLE-derived B cells exhibited significantly reduced IL-10 production following CD40 stimulation alone (Figure 5B). However, after MS5-3F coculture, both SLE and HC B cells efficiently expanded and differentiated into IL-10–producing populations, as confirmed by flow cytometry and ELISA of culture supernatants (Figure 5, C–E).

To assess the risk of IgG antibody production under these conditions, we next examined the isotype profile of secreted antibodies. In both SLE and HC samples, secreted antibodies were predominantly IgM, with minimal IgG detection (Figure 5F). This consistently IgM-dominant profile suggests that, under these conditions, unswitched plasmablast/plasma cells are preferentially expanded while IgG-secreting output remains limited, even in autoimmune contexts. Collectively, these results demonstrate that the MS5-3F platform enables robust expansion of IgM^+^ IL-10–producing plasmablasts from both HC and SLE-derived B cells, with minimal IgG secretion. Although the minimal IgG production observed may suggest a limited risk of expanding autoreactive clones, direct evaluation of autoreactivity will be required in future studies.

## Discussion

In this study, we developed a stromal coculture system using MS5 cells engineered to express human CD40L, BAFF, and IFN-β1 (MS5-3F), which enables efficient induction and robust expansion of human IL-10–producing B cells from peripheral blood. This system reproducibly generated high frequencies of IL-10–producing B cells with phenotypic and transcriptional features characteristic of unswitched (IgM^+^) plasmablasts. These cells demonstrated potent immunosuppressive activity, as evidenced by their ability to inhibit CD4^+^ T cell proliferation. Importantly, this approach was also effective in expanding IL-10–producing B cells derived from patients with SLE.

Compared with previously reported liquid culture methods using CpG, IL-2, IL-6, and type I IFN (9), our MS5-3F coculture platform offers key advantages, including support for long-term viability and expansion, thereby addressing critical limitations of previous approaches. Notably, it efficiently promotes differentiation from naive B cells into IgM^+^ IL-10–producing plasmablasts while limiting the expansion of IgG^+^ memory B cells. The mechanisms underlying this skewing remain incompletely understood but may involve a combination of accelerated differentiation kinetics and subset-intrinsic properties. IFN-β has been shown to suppress IgG class-switch recombination (CSR) (40) and our culture conditions lack cytokines that promote IgG switching, such as IL-4 and IL-21 (41–43). Moreover, early plasmablast differentiation may occur before CSR is completed; Blimp-1, a key transcription factor driving plasmablast commitment, is known to inhibit CSR (44). MS5 stromal cells may also contribute via adhesion-dependent or soluble signals that reinforce plasmablast survival, although this remains to be tested. Subset-intrinsic differences may also contribute. Our results further suggest that IL-10–producing B cells predominantly arise from naive B cells rather than memory B cells, even when both subsets are present at the start of culture. One possible explanation is that memory B cells rapidly differentiate into CD38^hi^CD27^hi^ plasma cells, bypassing the CD38^hi^CD27^lo^ plasmablast stage — the subset that showed the highest IL-10 production (9). In contrast, naive B cells may retain greater plasticity to adopt a regulatory phenotype under the influence of CpG, IFN-β, CD40L, and BAFF on MS5 stroma cells. Although direct evidence is limited, prior studies suggest that naive and memory B cells respond differently to key stimuli such as CpG, CD40L, BAFF, and type I IFNs, including differences in proliferation or differentiation (18, 45–52). These subset-specific differences, together with differential accessibility to transcriptional regulators such as STAT3, MAF, IRF4, and AhR, may underlie the divergent IL-10–inducing capacity observed. Further mechanistic studies will be necessary to clarify these pathways.

The functional relevance of the expanded IL-10–producing B cells was confirmed by their ability to suppress CD4^+^ T cell proliferation. This effect was largely dependent on IL-10, but not entirely abolished by IL-10 or IL-10R blockade, indicating possible contributions from IL-10–independent pathways (53). Molecules such as PD-L1 and TIM-1 have been implicated in IL-10–independent Breg function. PD-L1 expressed on B cells can directly inhibit T cell activation through engagement of PD-1 on T cells (54, 55), while TIM-1 signaling has been shown to promote regulatory B cell function and may act synergistically with IL-10 or independently (32, 56). Although we did not directly examine these pathways, they represent important directions for future study. In addition to suppressing proliferation, MS5-3F–expanded B cells increased the frequency of Tregs in vitro. Notably, this increase was not significantly affected by IL-10/IL-10R blockade, suggesting that Treg augmentation may occur through IL-10–independent mechanisms in this setting. Candidate pathways may include contact- and costimulation-related mechanisms (e.g., PD-L1, ICOS-L, or CD80/86) (39, 55, 57) and other regulatory cues; delineating the relative contributions of these mechanisms will be an essential topic for future work.

A further consideration is the cytokine heterogeneity of human IL-10^+^ B cells. In our system, IFN-γ and IL-12 were not detected, whereas a definable subset of IL-10^+^ cells coproduced IL-6 or TNF-α, consistent with previous reports of IL-10–producing B cell diversity (21, 35). Overall, these findings support an IL-10–oriented regulatory profile while underscoring the importance of monitoring cytokine coexpression in translational settings.

Notably, B cells from patients with SLE, despite impaired IL-10 induction under conventional conditions, efficiently differentiated into IL-10–producing plasmablasts in our system. These cells were largely IgM^+^ and showed minimal class switching and IgG secretion, suggesting a reduced risk of expanding autoreactive memory B cells. Nevertheless, a minor population of IgG^+^ cells was also observed, which could pose safety concerns depending on the clinical context and warrants careful monitoring in future applications. In addition, expansion efficiency varied across donors, potentially reflecting heterogeneity in immune status or treatment history. These factors should be taken into account when considering clinical application.

IL-10 has complex roles in SLE pathogenesis. In murine lupus models, IL-10 has been consistently shown to exert protective effects, as demonstrated by accelerated disease in IL-10–deficient mice (58–60), exacerbation upon in vivo IL-10R blockade (61), and disease amelioration following IL-10 overexpression (62). Although IL-10–producing B cell subsets such as CD24^hi^CD38^hi^ immature B cells are protective and often functionally deficient in patients with SLE (11, 24), IL-10 may also support the differentiation of autoreactive plasma cells, especially in the DN2 (CD27^–^IgD^–^T-bet^+^CD11c^+^CXCR5^–^) subset, which is considered a form of atypical memory B cell expanded in SLE and predominantly expresses IgG (63). Our system selectively expands naive-derived IL-10^+^IgM^+^ plasmablasts, which are phenotypically and functionally distinct from DN2 cells. This may mitigate the risk of exacerbating autoimmunity and improve the therapeutic index of IL-10–based approaches.

A practical consideration for cell-based delivery is both the durability of infused cells and their ability to traffic to lymphoid and disease-relevant tissues. In our system, plasmablast-to–plasma cell maturation continued through day 20, with concomitant increases in the absolute numbers of both CD38^hi^CD27^lo^ plasmablasts and CD38^hi^CD27^hi^ plasma cells. IL-10 expression remained enriched in the plasmablast compartment, while a definable subset of plasma cells retained IL-10 competence at later time points, suggesting that IL-10 production may extend beyond the earliest culture stage. In parallel, adhesion and chemokine receptor profiling suggest a phenotype compatible with access to both secondary lymphoid organs and inflamed tissues, potentially enabling encounters with CD4^+^ T cells across anatomical settings. Thus, these features are relevant for adoptive transfer, although direct in vivo validation will be required to define persistence, tissue distribution, and the durability of IL-10 output after transfer.

In conclusion, our stromal coculture system provides a robust and scalable platform for generating functional human IL-10–producing B cells. This approach enhances the regulatory potential of B cells, including those from autoimmune patients, while minimizing expansion of potentially pathogenic subsets. These findings provide a foundation for future development of B cell–based immunotherapies in autoimmune disease.

## Methods

### Sex as a biological variable.

Healthy male and female individuals were included in the study, and sex was not accounted for as a biological variable. Consistent with the female-dominant epidemiology of SLE, only female patients were enrolled in this study. Thus, our findings are applicable to the majority of patients with SLE, although their relevance to male patients requires further investigation.

### Human participants.

The study included adult HCs (aged 24–38 years) with no current medications, and female patients with SLE (aged 21–67 years) attending Kyushu University Hospital, all of whom had mild to moderate disease activity. PBMCs were isolated from both HCs and patients with SLE by density gradient centrifugation using Lympholyte Cell Separation Media – Human (Tebubio), according to the manufacturer’s instructions. Isolated PBMCs were cryopreserved and stored at –80°C until use in experiments.

### Generation of MS5-derived stromal cell lines.

To generate engineered stromal cell lines expressing human CD40L, BAFF, and IFN-β1, full-length cDNAs for CD40L, BAFF, and IFN-β1 were PCR amplified from MGC clones 30915276, 40002940, and 100004043, respectively, and cloned into the retroviral vectors pMY-IRES-DsRed2 (for CD40L), pMY-IRES-GFP (for BAFF), and pMX-puro (for IFN-β1). The pMY-IRES-DsRed2 vector was constructed by replacing the GFP in pMY-IRES-GFP with DsRed2. Retrovirus production was carried out by transfecting PLAT-E packaging cells with each construct using FuGENE HD (Promega) according to the manufacturer’s instructions. At 24 hours after transfection, the medium was replaced with fresh DMEM containing 10% (vol/vol) FBS (biosera), and viral supernatants were collected 48 hours later. Murine MS5 stromal cells, provided by Takafumi Yokota (Osaka University, Osaka, Japan), were transduced with viral supernatants in the presence of 2 μg/mL polybrene (Sigma-Aldrich) for 4 hours. For dual or triple transductions, spin infection was sequentially repeated with appropriate combinations of virus-containing supernatants. After infection, CD40L- or BAFF-expressing cells were sorted based on DsRed or GFP fluorescence, respectively. IFN-β1–expressing cells were selected using puromycin (4 μg/mL). To evaluate the expression level of IFN-β1, stromal cell lines were cultured for 24 hours, and the concentration of IFN-β1 in the supernatants was measured by ELISA. The resulting single-, dual-, and triple-transduced stromal cell lines were designated as MS5-CD40L, MS5-CD40L/BAFF, MS5-IFNβ1, and MS5-3F (expressing CD40L, BAFF, and IFN-β1), respectively, and were used in subsequent experiments. Established lines were maintained in MEMα medium (Wako) supplemented with 10% (vol/vol) FBS (Biosera), 1% penicillin/streptomycin, and 2 mM L-glutamine.

### Preparation of human B cells and T cells.

Primary human B cells were isolated from PBMCs by positive selection using CD19 MicroBeads (Miltenyi Biotec). In some experiments, enriched B cells were further purified by FACSMelody (BD Biosciences) cell sorter. For T cell purification, CD19^+^ B cells and CD8^+^ T cells were first depleted using CD19 and CD8 MicroBeads (Miltenyi Biotec). CD4^+^ T cells were then negatively selected by depleting monocytes and NK cells using the MicroBeads cocktail included in the CD4^+^CD25^+^ Regulatory T Cell Isolation Kit (Miltenyi Biotec). CD4^+^CD25^–^ naive T cells and CD4^+^CD25^+^ Tregs were subsequently isolated from the CD4^+^ T cell fraction using CD4^+^CD25^+^ Regulatory T Cell Isolation Kit (Miltenyi Biotec). The purity of isolated B and T cell populations was routinely assessed by flow cytometry, and only samples with 95% or greater purity were used in downstream experiments.

### Human B cell coculture with MS5 stromal cells.

Human B cells (3 × 10^4^) were cocultured with MS5 stromal cells (5 × 10^3^) in 96-well round-bottom plates using MEMα medium supplemented with 10% (vol/vol) GemCell Human AB Serum (Gemini Bio-Products), 10% (vol/vol) FBS (Capricorn Scientific), 1% MEM Non-Essential Amino Acids, 1% Penicillin-Streptomycin, and 2 mM L-glutamine (Nacalai Tesque). In some experiments, the culture medium was further supplemented with 1 μg/mL class B CpG oligonucleotide 2006 (ODN 7909, InvivoGen), 100 ng/mL recombinant human IL-2, 100 ng/mL recombinant human IL-6 (R&D Systems), and 1000 U/mL Universal Type I IFN-α (PBL Assay Science). On day 4, nonadherent (floating) B cells were collected, resuspended in fresh medium, and transferred to 96-well flat-bottom plates seeded with fresh MS5 cells (5 × 10^3^ cells per well). On day 8, the cells were again collected, resuspended, and divided into 3 aliquots. Each aliquot was transferred to a new flat-bottom plate containing freshly plated MS5 cells. This passage procedure was repeated every 4 days to maintain cell expansion and viability. To assess the IL-10 production capacity of B cells prior to coculture, human B cells (1 × 10^5^) were stimulated with CD40L-expressing MS5 cells (5 × 10^3^) in 96-well round-bottom plates for 2 days in the absence of CpG.

### B cell culture under proinflammatory conditions.

To evaluate IL-10 production stability under inflammatory conditions, B cells were cocultured with MS5-3F, harvested on day 12, and then subjected to additional liquid culture for 4 days with supplementation of 70 pg/mL IFN-α (PBL Assay Science), 20 pg/mL recombinant human IFN-γ (BioLegend), 76 pg/mL recombinant human IL-12 p70 (BioLegend), 33 pg/mL recombinant human IL-6 (BioLegend), and 12 pg/mL recombinant human TNF-α (BioLegend). To assess the concentration-dependent effects of these cytokines, 4 conditions were tested: ×1 (high), ×1/4 (medium), ×1/16 (low), and ×0 (without cytokines). After additional culture, IL-10 production was assessed by an IL-10 secretion assay.

### B cell restimulation and T cell proliferation assay.

Human B cells cocultured with MS5-3F for 12 days were harvested using density gradient centrifugation with Percoll (GE Healthcare Life Sciences). Recovered B cells were restimulated in culture medium containing 20 μg/mL F(ab′)_2_ fragment goat anti–human IgM (Jackson ImmunoResearch, 109-006-129) and 1 μg/mL CpG ODN 2006 (InvivoGen) at a concentration of 9 × 10^5^ cells/mL. The cells were cultured for 2 days at 37°C in a 5% CO_2_ incubator. To evaluate the suppressive capacity of primary B cells, restimulated B cells, and autologous CD4^+^CD25^+^ Tregs, CD4^+^CD25^–^ naive T cells were labeled with a CellTrace Violet Cell Proliferation Kit (Thermo Fisher Scientific) and seeded at 2.5 × 10^5^ cells/mL in the presence of plate-bound anti–human CD3 (BioLegend, 317325, clone: OKT3, 1 μg/mL) and anti–human CD28 (BioLegend, 302933, clone: CD28.2, 1 μg/mL) antibodies. T cells were then cultured with/without an equal number of primary B cells, restimulated B cells, or Tregs for 5 days. In some experiments, primary B cells and restimulated B cells were added at varying B/T ratios as indicated in the figure legends. The inhibitory efficiency of T cell proliferation by B cells and Tregs (%inhibition) were calculated as follows: %inhibition = (1 − [division index of experimental group/division index of control]) × 100. In some experiments, neutralizing or isotype control antibodies were added to the cultures at a final concentration of 10 μg/mL, including anti–human IL-10 (501427, clone: JES3-9D7), anti–human IL-10R (308817, clone: 3F9), rat IgG1, κ isotype control (400431, clone: RTK2071), and rat IgG2a, κ isotype control (400501, clone: RTK2758) (all from BioLegend). To evaluate Treg induction, CD4^+^CD25^–^ T cells were cultured with/without primary B cells or MS5-3F–induced B cells in the presence of plate-bound anti–human CD3 antibody (BioLegend, 317325, clone: OKT3, 1 μg/mL) for 3 days.

### Flow cytometry and antibodies.

For surface staining, MS5 and human cells were first incubated with polyclonal IgG (Santa Cruz Biotechnology, sc-2025) or Fc Receptor Binding Inhibitor (Thermo Fisher Scientific, 14-9161-73), respectively, for 10 minutes on ice to block nonspecific binding. Cells were then stained with fluorochrome-conjugated antibodies specific for surface antigens for 20 minutes on ice. After staining, cells were washed twice with staining buffer and analyzed using a CytoFLEX S flow cytometer (Beckman Coulter). Dead cells were excluded using propidium iodide (Nacalai Tesque) for T cell proliferation assays or 7-AAD (BD Biosciences) for other surface staining experiments. To assess IL-10 secretion by B cells, the cells were stimulated with 50 ng/mL PMA (Nacalai Tesque) and 1 μg/mL ionomycin (Sigma-Aldrich) for 3 hours. IL-10 secretion was detected using the IL-10 Secretion Assay – Detection Kit (APC, Miltenyi Biotec), according to the manufacturer’s instructions. For intracellular transcription factor staining, the eBioscience Foxp3/Transcription Factor Staining Buffer Set (Thermo Fisher Scientific) was used following the manufacturer’s protocol. Dead cells were excluded using the Zombie Aqua Fixable Viability Kit (BioLegend).

The following antibodies were purchased from BD Biosciences: Alexa Fluor 647–conjugated anti–Blimp-1 (565002, clone: 6D3), rat IgG2a, κ (557690, clone: R35-95), Brilliant Violet 421–conjugated anti-CD19 (562441, clone: HIB19), Brilliant Violet 605–conjugated anti-CXCR4 (740418, clone: 12G5), mouse IgG2a, κ (562778, clone: G155-178), PerCP-Cy 5.5–conjugated anti-CD4 (552838, clone: L200), PE-conjugated anti-CD27 (560609, clone: M-T271), anti-CD180 (551953, clone: G28-8), and anti–granzyme B (561142, clone: GB11). The following antibodies were purchased form BioLegend: Alexa Fluor 647–conjugated anti-CD24 (311110, clone: ML5), anti-Pax5 (649703, clone: 1H9), anti-IRF4 (646407, clone: IRF4.3E4), anti-CD62L (304818, clone: DREG-56), rat IgG2a, κ (400526, clone: RTK2758), rat IgG1, κ (400418, clone: RTK2071), APC-conjugated anti-CD4 (344613, clone: SK3), anti-BAFF (366507, clone: 1D6), anti–IL-6 (501112, clone: MQ2-13A5), anti–IL-12/IL-23 p40 (501809, clone: C11.5), anti-CD49d (304307, clone: 9F10), anti-CTLA4 (349907, clone: L3D10), rat IgG1, κ (400418, clone: RTK2071), mouse IgG1, κ (400119, clone: MOPC-21), APC-Cy7–conjugated anti-CD19 (302217, clone: HIB19), anti-CCR5 (359110, clone: J418F1), rat IgG2b, κ (400623, clone: RTK4530), Brilliant Violet 421–conjugated anti-CD38 (303525, clone: HIT2), anti-CD40L (310823, clone: 24-31), anti-CD127 (351309, clone: A019D5), anti-IgM (314521, clone: MHM-88), anti-IgD (348225, clone: IA6-2), anti-CXCR3 (353715, clone: G025H7), mouse IgG1, κ (400157, clone: MOPC-21), Brilliant Violet 605–conjugated anti-CD192 (357213, clone: K036C2), Brilliant Violet 650–conjugated anti-CCR7 (353234, clone: G043H7), FITC-conjugated anti-CD8a (301005, clone: RPA-T8), anti-CD25 (356105, clone: M-A251), anti-CD29 (303015, clone: TS2/16), anti-CD38 (303504, clone: HIT2), anti–TNF-α (502906, clone: MAb11), anti-CXCR5 (356913, clone: J252D4), anti–IgG Fc (410719, clone: M1310G05), mouse IgG1, κ (400107, clone: MOPC-21), PE-conjugated anti-CD27 (356406, clone: M-T271), anti-CD54 (353105, clone: HA58), anti–TGF-β1 (300003, clone: S20006A), anti–IFN-γ (502510, clone: 4S.B3), anti-CD180 (312906, clone: RP105), anti-FOXP3 (320107, clone: 206D), mouse IgG1, κ (400111, clone: MOPC-21), PE/Dazzle 594–conjugated anti–IL-10 (506811, clone: JES3-19F1), rat IgG2a, κ (400557, clone: RTK2758), PE-Cy7–conjugated anti-IgD (348225, clone: IA6-2), anti-CD19 (302216, clone: HIB19), anti-CD20 (302311, clone: 2H7), anti-CD44 (103029, clone: IM7), anti-CD138 (356513, clone: MI15), rat IgG2b, κ (400617, clone: RTK4530), and biotin-conjugated anti-CD20 (302350, clone: 2H7). Biotin-conjugated antibody was detected with Streptavidin-BV510 (BioLegend). Flow cytometry data were analyzed using FlowJo (FlowJo, LLC).

### ELISA.

Human IFN-β production by MS5 cells and IL-10 secretion by B cells were quantified using the Verikine Human Interferon Beta ELISA Kit (PBL Assay Science) and the Human IL-10 ELISA MAX Deluxe Set (BioLegend), respectively, according to the manufacturers’ protocols. The concentrations of IgM and IgG in culture supernatants were measured using a custom sandwich ELISA. For capture antibodies, mouse anti–human IgM (555780, clone: G20-127) and mouse anti–human IgG (555784, clone: G18-145) (both from BD Biosciences) were used. For HRP-conjugated detection, mouse anti–human IgM (9022-05, clone: UHB) and mouse anti–human IgG Fc (9042-05, clone: H2) (both from Southern Biotech) were employed. Human IgM, myeloma (Rockland, 009-0107-0001) and purified human IgG (Bethyl Laboratories, P80-105) were used to generate standard curves. In brief, culture supernatants were collected and applied to plates precoated with capture antibodies. After washing, HRP-conjugated detection antibodies were added. Signal was developed using SureBlue TMB substrate (KPL), and absorbance was measured at 450 nm using a microplate reader (iMArk Microplate Reader, Bio-Rad). Protein concentrations were determined by comparison to the corresponding standard curves.

### Statistics.

All values are expressed as mean ± SEM. For comparisons of 2 groups, a 2-tailed, unpaired Student’s *t* test or the Welch’s *t* test was used. For multiple comparisons, 1-way analysis of variance (ANOVA) with Tukey’s multiple-comparison test or 2-way ANOVA with Bonferroni’s correction was used. *P* values of less than 0.05 were considered statistically significant. All statistical analyses were performed using Prism (GraphPad Software).

### Study approval.

Written informed consent was obtained from all participants in accordance with the Declaration of Helsinki. The study protocol was approved by the Institutional Review Board of Kyushu University Hospital (approval no. 2023-51 and 2022-195).

### Data availability.

Individual values for all data points shown in the figures are provided in the Supporting Data Values file.

## Author contributions

Conceptualization, original hypothesis, and design of the study: YB. Methodology: RK and YB. Investigation: RK, KI, AB, YS, KK, YY, RH, AS, RI, SP, RK, HN, ST, and YB. Writing – original manuscript draft: RK and YB. Writing – review & editing: RK, KI, AB, YS, KK, YY, RH, AS, RI, SP, RK, HN, ST, and YB. Resources: HN, KI, YS, YY, and RH. Supervision: YB.

## Conflict of interest

The authors declare that no conflict of interest exists.

## Funding support

- Japan Society for the Promotion of Science KAKENHI (JP16K15217, JP18H02626, JP21K18256, JP21H02753, and JP24K02297) to YB.
- Japan Agency for Medical Research and Development (AMED) (JP20gm6110004, JP24gm0010010, JP23gm1810008, and JP24gm1810008) to YB.
- MEXT Promotion of Development of a Joint Usage/Research System Project to YB.
- Medical Research Center Initiative for High Depth Omics and RIIT to YB.
- MEXT Promotion of Development of a Joint Usage/Research System Project: Coalition of Universities for Research Excellence Program (CURE) grant number JPMXP1323015486 to YB.

## Supplementary Material

Supplemental data

Supporting data values

## Version 1 04/22/2026

Electronic publication
