# Supplementary material for: A stromal platform for robust expansion of functional IL-10–producing B cells for immune regulation
Source: JCI Insight. 2026 Apr 22;11(8):e197393. doi: 10.1172/jci.insight.197393 (PMC13135411; doi:10.1172/jci.insight.197393)
Supplement: Supplemental data [file jciinsight-11-197393-s043.pdf]

SUPPLEMENTAL MATERIAL

A stromal platform for robust expansion of functional IL-10–producing B cells for immune regulation

Ryo Kawakami, Keisuke Imabayashi, Akemi Baba, Yuichi Saito, Kazuhiko Kawata, Yutaro Yada, Airi Shibata, Rinka Ito, Ryo Kurasawa, Ryota Higuchi, Sungyeon Park, Hiroaki Niiro, Shinya Tanaka, Yoshihiro Baba

Supplemental Figure 1. Confirmation of CD40L and BAFF expression on MS5, related to Figure 1.

Supplemental Figure 2. Optimization of co-culture conditions for the induction and expansion of human IL-10–producing B cells, related to Figure 1.

Supplemental Figure 3. Kinetics of plasmablast differentiation in the MS5 co-culture system, related to Figure 2.

Supplemental Figure 4. Evaluation of the autocrine effects of IL-10 in the MS5-3F co-culture system, related to Figure 2.

Supplemental Figure 5. Day 20 dynamics of plasmablast/plasma-cell accumulation and IL-10 competence, related to Figure 3.

Supplemental Figure 6. Sorting strategy for isolation of memory, naive mature, and naive immature B cells, related to Figure 3.

Supplemental Figure 7. Cytokine production by MS5-3F–induced B cells, related to Figure 3.

Supplemental Figure 8. Expression of chemokine receptors and adhesion molecules by MS5-3F–induced B cells (day 12), related to Figure 3.

Supplemental Figure 9. Stability of IL-10 production by MS5-3F–induced B cells under pro-inflammatory condition, related to Figure 3.

Supplemental Figure 10. Suppressive mechanism of MS5-3F–induced IL-10–producing B cells, related to Figure 4.

Supplemental Figure 11. Confirmation of CD40L expression on MS5, related to Figure 5.

Supplemental Table 1. Clinical data of SLE patients in experiments, related to Figure 5.

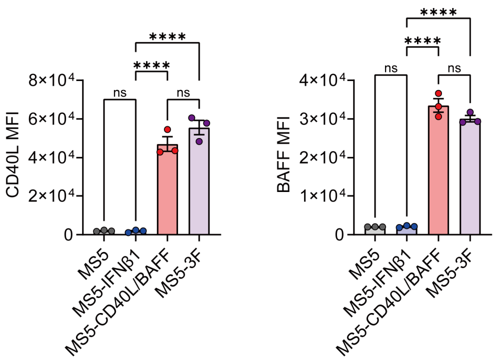

Supplemental Figure 1. Confirmation of CD40L and BAFF expression on MS5, related to Figure 1.

MFI values of human CD40L and BAFF expressed on engineered MS5. Data from three independent experiments were combined (n = 3). Data are presented as mean ± SEM. P values are from one-way ANOVA with post-hoc Tukey HSD test. ****$P \leq 0.0001$; ns, not significant.

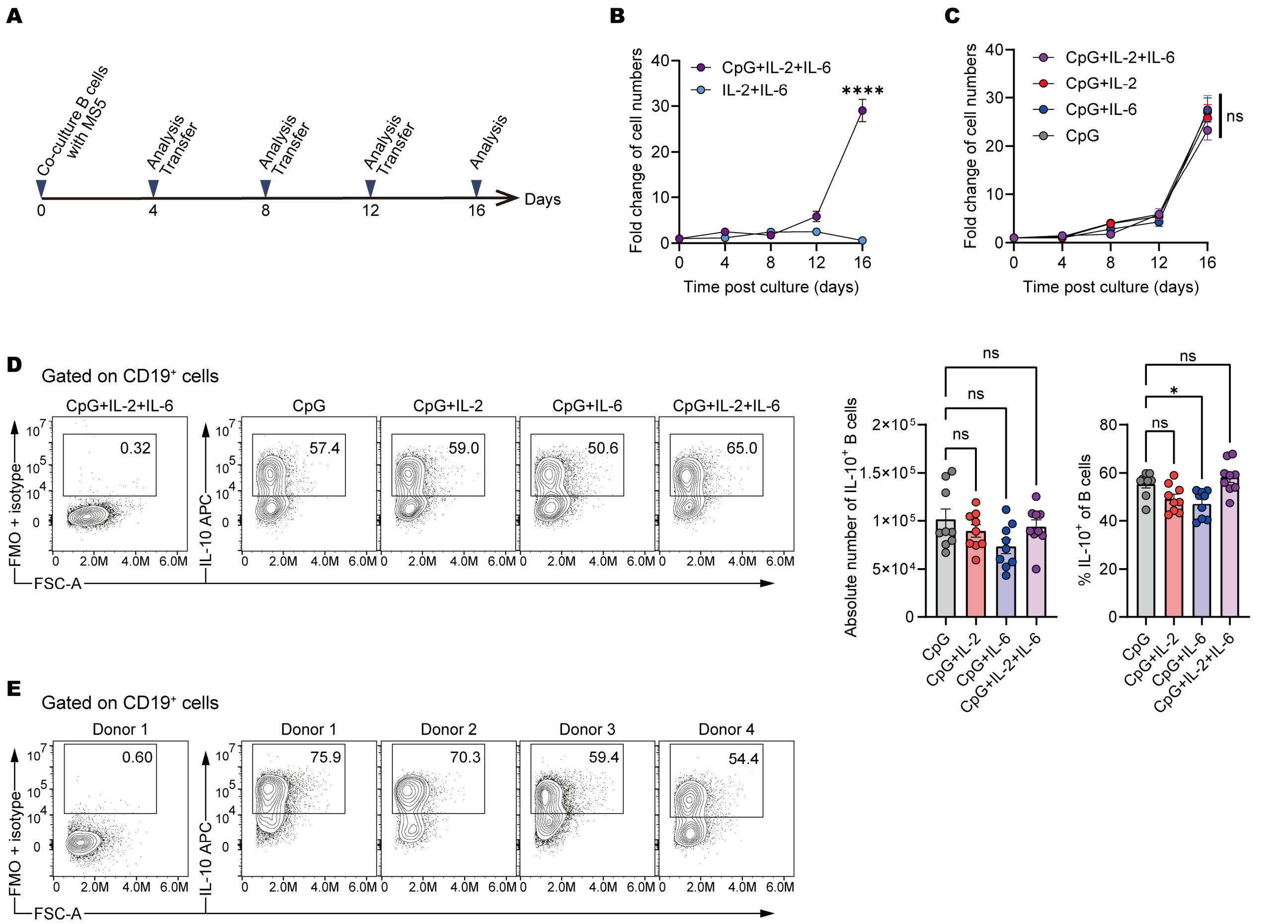

Supplemental Figure 2. Optimization of co-culture conditions for the induction and expansion of human IL-10–producing B cells, related to Figure 1.

(**A**) Schematic of co-culture. (**B**) Expansion of human B cells co-cultured with MS5-3F in the presence or absence of CpG-ODN. Data from three independent experiments were combined (n = 3). (**C**) Expansion of human B cells co-cultured with MS5-3F in the presence or absence of IL-2 and/or IL-6. Data from three independent experiments were combined (n = 9). (**D**) Induction and expansion of IL-10–producing B cells at day 12 of co-culture with MS5-3F in the presence or absence of IL-2 and/or IL-6. Data from three independent experiments were combined (n = 9). (**E**) Representative flow cytometry plots of IL-10 in B cells from different donors on day 12 of MS5-3F co-culture. Data are presented as mean ± SEM. P values are from one-way ANOVA with post-hoc Tukey HSD test (**D**) or two-way ANOVA with Bonferroni correction (**B** and **C**). $^{*}P \leq 0.05$, $^{****}P \leq 0.0001$; ns, not significant.

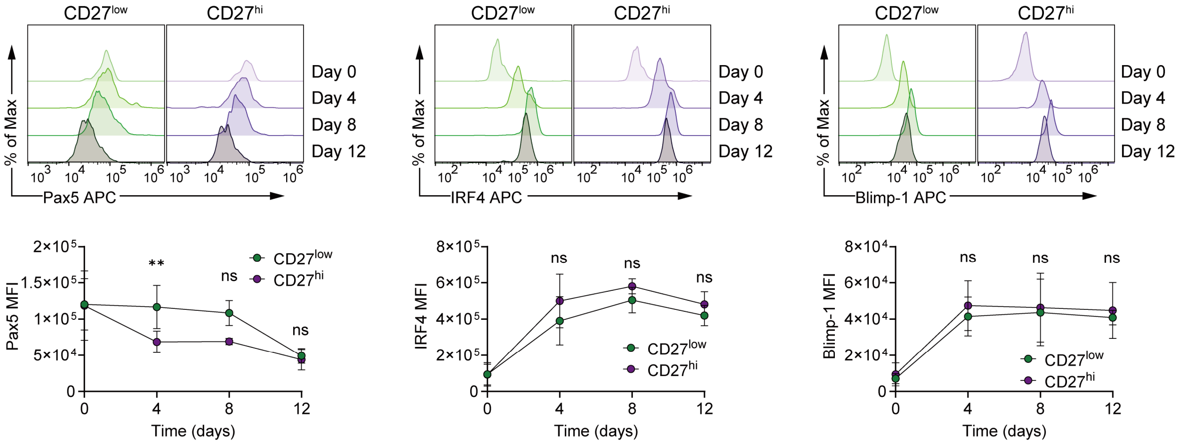

Supplemental Figure 3. Kinetics of plasmablast differentiation in the MS5 co-culture system, related to Figure 2.

Representative flow cytometry plots and time course of transcription factors defining plasmablasts and plasma cells expressed by MS5-3F–induced CD27^{low} and CD27^{hi} B cells at the indicated days of culture. Data from three independent experiments were combined (n = 3). Data are presented as mean ± SEM. P values are from two-tailed unpaired Student's t test. *$P \le 0.05$, ****$P \le 0.0001$; ns, not significant.

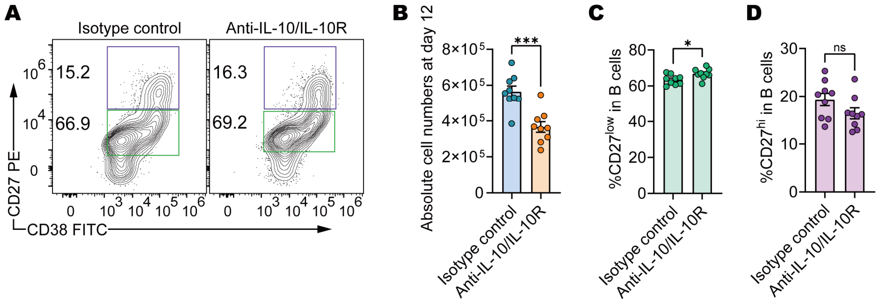

Supplemental Figure 4. Evaluation of the autocrine effects of IL-10 in the MS5-3F co-culture system, related to Figure 2.

(**A**–**D**) Analysis of B cells co-cultured with MS5-3F for 12 days in the presence of anti-IL-10/IL-10R or isotype control antibodies. (**A**) Representative flow cytometry plots of activation markers. (**B**) Total cell number. (**C**) Frequency and absolute number of CD38^{hi}CD27^{low} B cells. (**D**) Frequency and absolute number of CD38^{hi}CD27^{hi} B cells. Data from three independent experiments were combined (n = 9). Data are presented as mean ± SEM. P values are from two-tailed unpaired Student's t test. *$P \leq 0.05$, ***$P \leq 0.001$; ns, not significant.

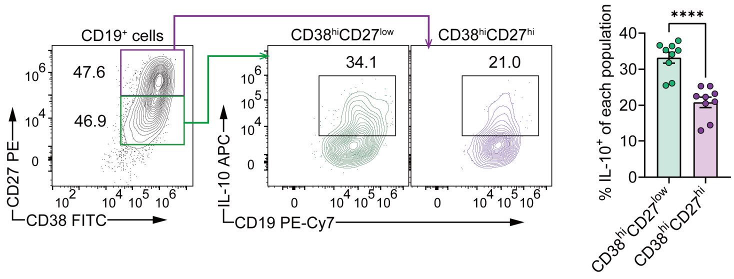

Supplemental Figure 5. Day 20 dynamics of plasmablast/plasma-cell accumulation and IL-10 competence, related to Figure 3.

Frequencies of IL-10$^+$ cells within CD27^{hi} and CD27^{low} subsets on day 20 of co-culture. Data from three independent experiments were combined (n = 9). Data are presented as mean ± SEM. P values are from two-tailed unpaired Student's t test. ****$P \leq 0.0001$.

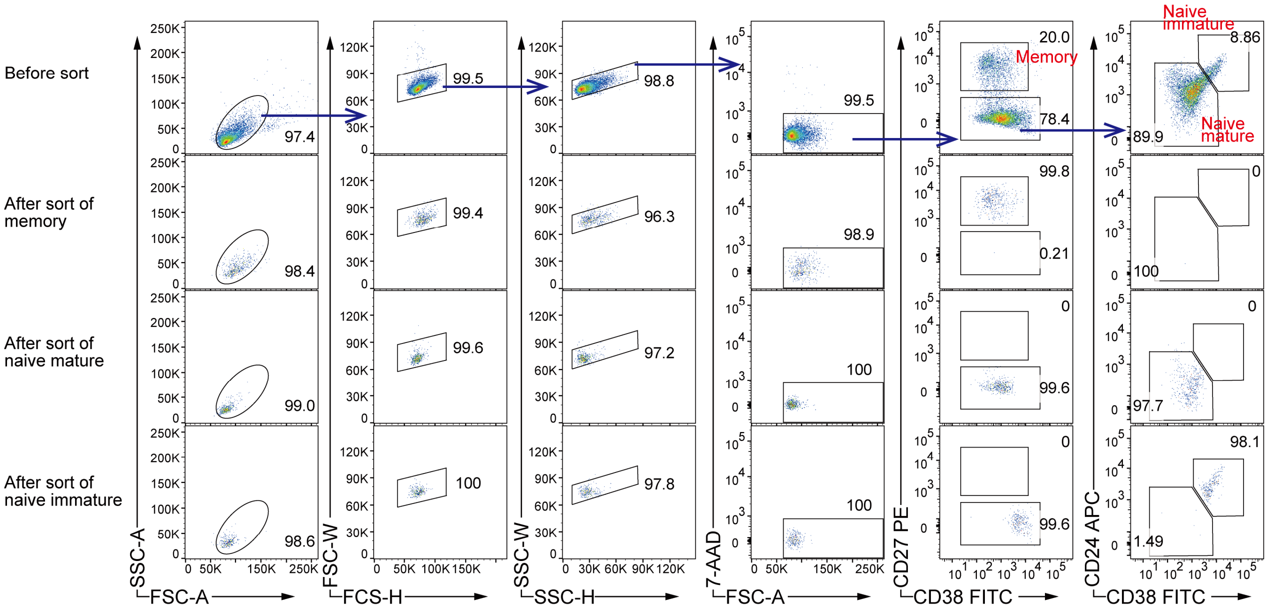

Supplemental Figure 6. Sorting strategy for isolation of memory, naive mature, and naive immature B cells, related to Figure 3.

Human B cells were first enriched from peripheral blood mononuclear cells (PBMCs) by positive selection with CD19 MicroBeads, achieving a purity of >98%. For flow cytometric sorting, lymphocytes were gated on the basis of forward and side scatter properties, followed by the exclusion of doublets and dead cells. Memory ($CD27^{+}$) and naive B cells ($CD27^{-}$) were then distinguished by CD27 expression, and within the naive compartment, mature ($CD38^{-}CD24^{+}$) and immature ($CD38^{+}CD24^{hi}$) fractions were sorted according to differential CD38 and CD24 expression levels.

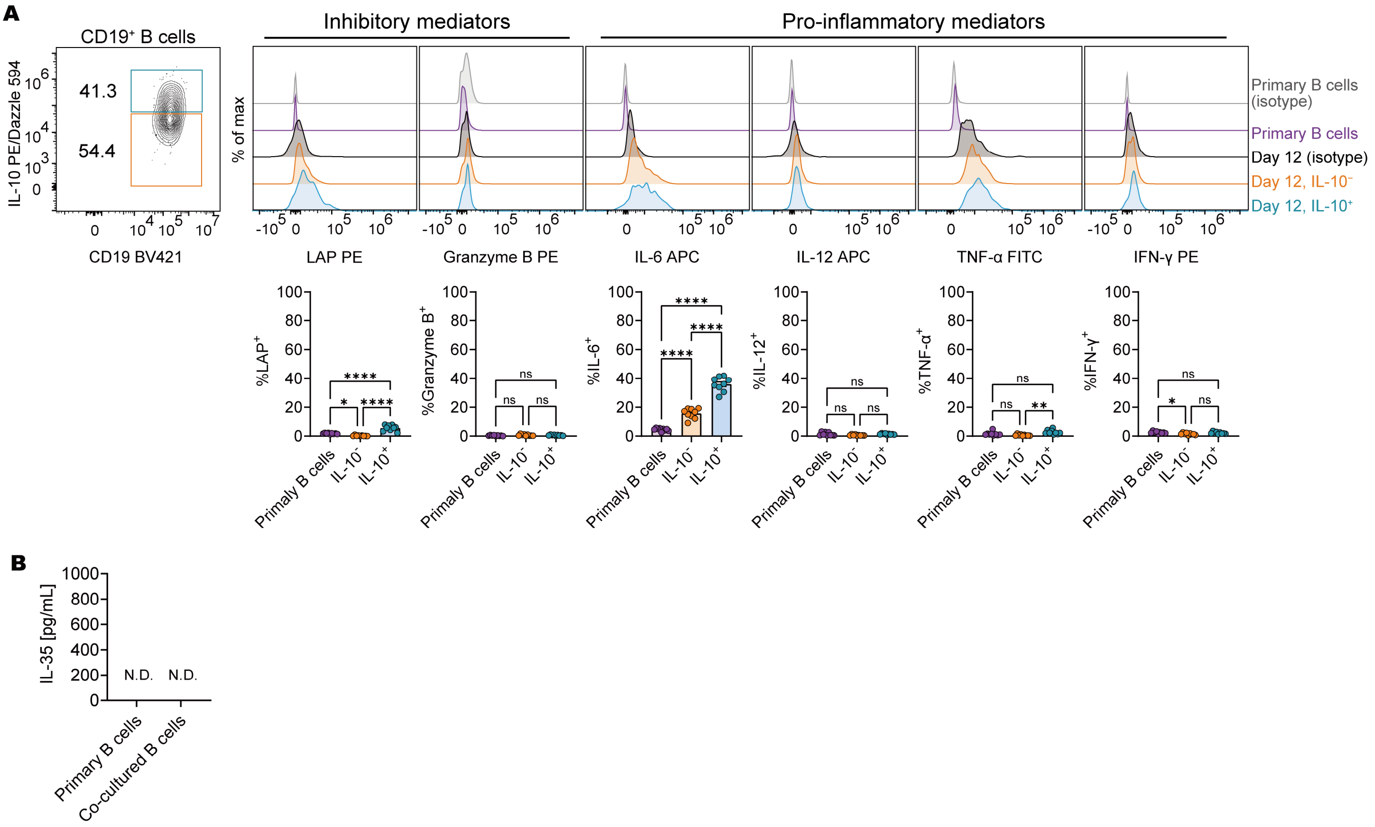

Supplemental Figure 7. Cytokine production by MS5-3F–induced B cells, related to Figure 3. (**A**) Representative flow cytometry histograms and summarized graphs showing inflammatory and regulatory molecules production by primary B cells and IL-10$^+$ and IL-10$^-$ MS5-3F–induced B cells (day 12). For each cytokine, positive gates were set based on matched isotype controls, and cytokine-positive frequencies were quantified accordingly. (**B**) IL-35 concentrations in culture supernatants measured by ELISA. Data from three independent experiments were combined (n = 9). Data are presented as mean ± SEM. P values are from one-way ANOVA. *$P \leq 0.05$, **$P \leq 0.01$, ****$P \leq 0.0001$; ns, not significant; N.D., not detected.

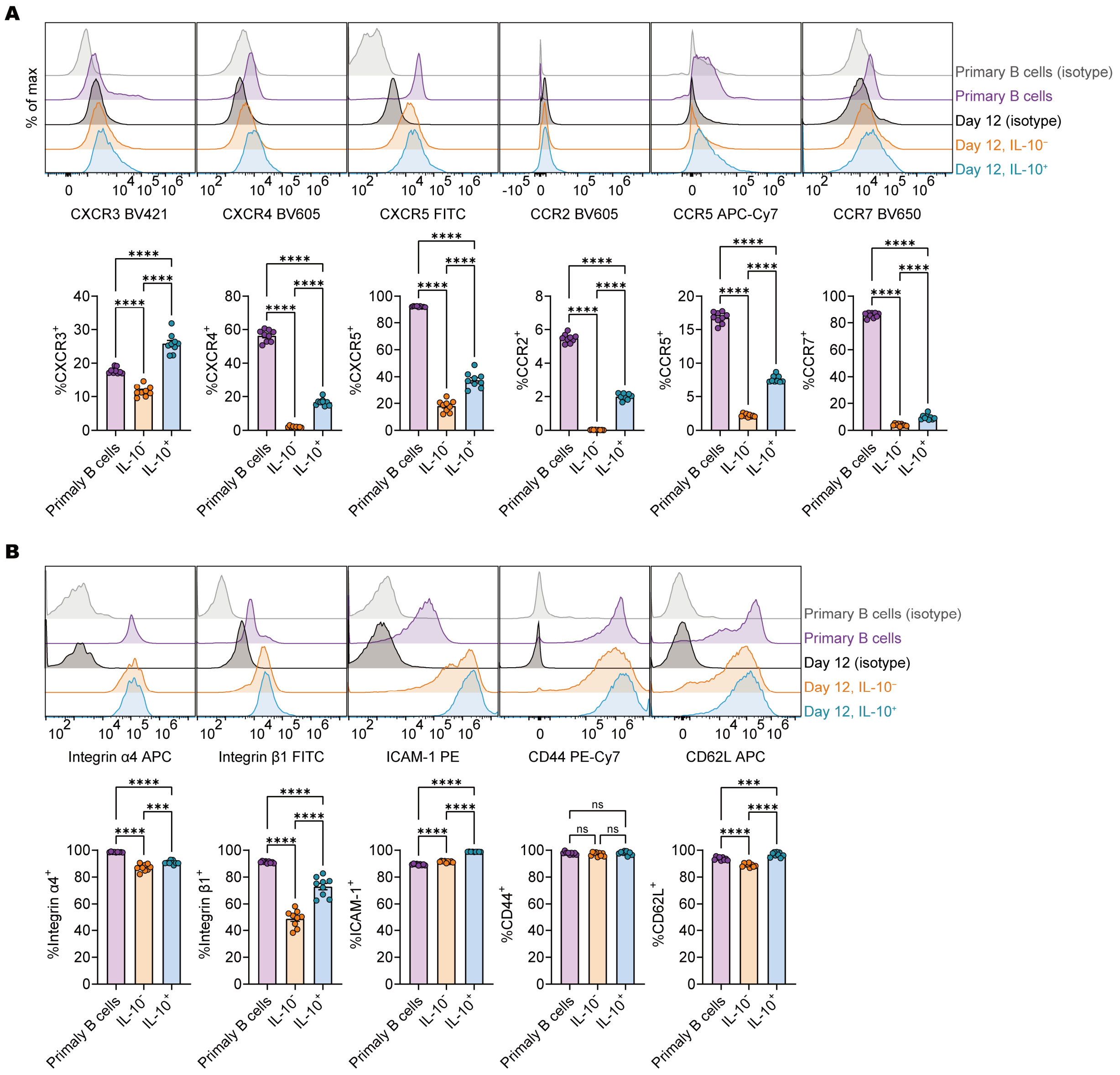

Supplemental Figure 8. Expression of chemokine receptors and adhesion molecules by MS5-3F–induced B cells (day 12), related to Figure 3.

(**A**) Representative flow cytometry histograms and summarized graphs showing chemokine receptors expressed by primary B cells and IL-10$^+$ and IL-10$^-$ MS5-3F–induced B cells. (**B**) Representative flow cytometry histograms and summarized graphs showing adhesion molecule expressed by primary B cells and IL-10$^+$ and IL-10$^-$ MS5-3F–induced B cells. Data from three independent experiments were combined (n = 9). Data are presented as mean ± SEM. P values are from one-way ANOVA. ***$P \leq 0.001$, ****$P \leq 0.0001$; ns, not significant.

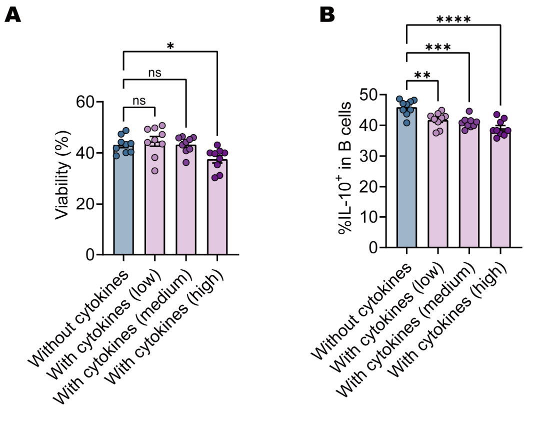

Supplemental Figure 9. Stability of IL-10 production by MS5-3F–induced B cells under pro-inflammatory condition, related to Figure 3.

B cells were co-cultured with MS5-3F, harvested on day 12, and then re-cultured in liquid medium for an additional 4 days either without cytokines or with a pro-inflammatory cytokine cocktail. The "high" cocktail contained IFN-α (70 pg/mL), IFN-γ (20 pg/mL), IL-12p70 (76 pg/mL), IL-6 (33 pg/mL), and TNF-α (12 pg/mL); the "medium" and "low" conditions represent 1/4× and 1/16× dilutions of this high cocktail, respectively. After re-culture, cell viability was assessed by dead-cell staining (**A**), and IL-10 production was evaluated using an IL-10 secretion assay (**B**). Data from three independent experiments were combined (n = 9). Data are presented as mean ± SEM. P values are from one-way ANOVA with post-hoc Tukey HSD test. *$P \leq 0.05$, **$P \leq 0.01$, ***$P \leq 0.001$, ****$P \leq 0.0001$; ns, not significant.

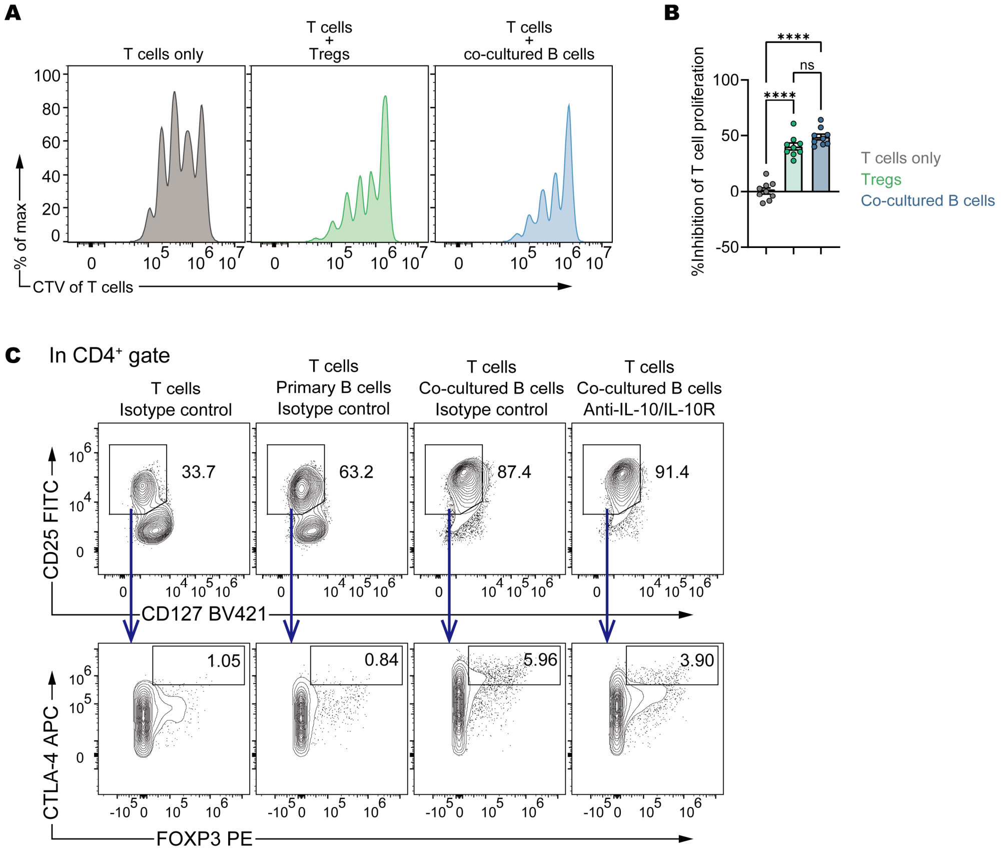

Supplemental Figure 10. Suppressive mechanism of MS5-3F–induced IL-10–producing B cells, related to Figure 4.

(**A** and **B**) Comparison of suppressive efficacy between MS5-3F–induced B cells and autologous $CD4^+CD25^+$ Tregs. (**A**) Representative flow cytometry histograms of CTV-labeled $CD4^+$ T cells are shown for the three culture conditions: T cells alone, T cells co-cultured with $CD4^+CD25^+$ Tregs, and T cells co-cultured with MS5-3F–induced B cells. (**B**) Summarized %inhibitions of T cell proliferation. (**C**) Representative flow cytometry plots showing Treg induction by primary B cells and MS5-3F–induced co-cultured B cells. Data from three independent experiments were combined ($n = 9$). Data are presented as mean ± SEM. P values are from one-way ANOVA with post-hoc Tukey HSD test. ****$P \leq 0.0001$; ns, not significant.

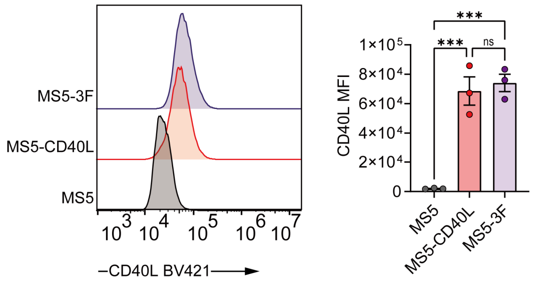

Supplemental Figure 11. Confirmation of CD40L expression on MS5, related to Figure 5. Representative flow cytometry histograms showing surface expression of human CD40L on engineered MS5 stromal cells, and MFI values of human CD40L expressed on MS-5. Data from three independent experiments were combined ($n = 3$). Data are presented as mean ± SEM. P values are from one-way ANOVA with post-hoc Tukey HSD test. ***$P \leq 0.001$; ns, not significant.

Supplemental Table 1. Clinical data of SLE patients in experiments, related to Figure 5.

Subject	Gender	Age	Past symptom	Symptom at the day of venipuncture	Therapies	SLENA-SLEDAI	Autoantibody	Anti-dsDNA [IU/mL] (Normal: <10)	C3 [mg/dL] (Normal: 73 – 138)	C4 [mg/dL] (Normal: 11 – 31)
1	F	50	arthralgia, cytopenia, pulmonary hypertension	none	PSL, TAC	2	SSA, cardiolipin	3.9	87	9
2	F	21	arthritis, fatigue	arthritis, fatigue	HCQ	10	Sm, SSA, SSB, RNP, dsDNA	337.9	29	2
3	F	57	fatigue, arthralgia, cytopenia	fatigue, arthralgia, cytopenia	PSL, HCQ, BLM	8	none	0.6	115	7
4	F	67	skin rash, pleuritis	skin rash	HCQ, BLM	4	RNP, dsDNA	1258.5	74	14

PSL: prednisolone, TAC: tacrolimus, HCQ: hydroxychloroquine, BLM: belimumab
